# The relationship between vitamin D and spontaneous abortion among Iraqi women

**DOI:** 10.25122/jml-2021-0266

**Published:** 2022-06

**Authors:** Sumayah Faruq Kasim

**Affiliations:** 1 College of Health and Medical Technologies, Middle Technical University, Baghdad, Iraq

**Keywords:** miscarriage, abortion, vitamin D, pregnancy, vitamin D deficiency

## Abstract

Miscarriage is the most common complication of pregnancy. Moreover, vitamin D deficiency is a prevalent concern among women of reproductive age, particularly in the Arab world, where the link between vitamin D deficiency and miscarriage is still unknown. This study was conducted to determine the relationship between vitamin D and miscarriage by comparing the concentration of vitamin D among women with spontaneous abortion and pregnant women. A total of 80 subjects were enrolled in this study and divided into two groups. The first group included 40 women with spontaneous abortions aged between 18 and 40 years. The second group included 40 pregnant women without previous history of miscarriages. Total 25-hydroxy vitamin D (25-OH-VD) measurement was estimated with a Dry Fluorescence Immunoassay analyzer using the Lansionbio LS-1100 instrument. The relationship between the five age groups and the vitamin D status of women with spontaneous abortion was not significant (p>0.05). There was no significant relationship between the miscarriage trimesters and vitamin D status (p>0.05) and between the five age groups (p>0.05). In our study, 95% of women with spontaneous abortion had vitamin D deficiency, compared to only 17.5% of pregnant women. A normal range of vitamin D improves the growth of the fetus and prevents pregnancy complications and miscarriage, promoting the growth of blood vessels in the placenta and improving the function of immune cells.

## INTRODUCTION

Globally, reproductive health is a major concern, especially for women of childbearing age [1]. Miscarriage is one of the issues that can occur during pregnancy and impact reproductive health. As the name suggests, it refers to the loss of an embryo before the twentieth week of pregnancy. Spontaneous abortion is the medical word for a miscarriage. Abortion can be classified as early and late abortion (<12 weeks and ≥28 weeks) [2]. Miscarriage can be caused by various factors, including chromosomal abnormalities, uterine abnormalities, hormone issues, infections, immunological disorders, and thrombophilia, and up to 50% of cases have no obvious cause [3].

Vitamin D insufficiency is a modified risk factor for miscarriage. Recent studies showed that vitamin D levels are significantly lower than needed to sustain health. A vitamin D deficit was seen in tropical nations despite the high exposure to UV light [4]. Vitamin D has a well-established role in regulating calcium homeostasis and promoting bone mineralization as a steroid hormone [5]. Recently, vitamin D nuclear receptors were found in a variety of tissues, including reproductive and infant-growing organs, the ovary, testis, placenta, and mammary gland [6].

Vitamin D deficiency during pregnancy is a worldwide pandemic, with prevalence ranging from 18 to 84 percent, where most pregnant females had vitamin D deficiency, and 10% had normal levels. This revealed that the prevalence of vitamin D deficiency among pregnant women reached 40% and 28.9%, respectively [7]. Multiple risk factors affect hypovitaminosis among pregnant women: air pollution, high latitude, seasonal variation in winter and autumn and the duration of sun exposure, obesity, black skin color, low education level, and low income of the mother. In addition, preeclampsia, gestational diabetes, bacterial vaginosis, and impaired intrauterine development have also been linked to low levels of vitamin D during pregnancy [7].

According to some research, preterm delivery is related to vitamin D insufficiency during pregnancy [8]. However, the link between vitamin D deficiency and insufficiency in the first trimester and miscarriage or spontaneous pregnancy loss in childbearing women is less apparent. There are also many enzyme processes involved in the vitamin D metabolism pathway. This hormone is also a risk factor for miscarriage since it is synthesized in the human placenta and deciduas [8]. However, low vitamin D levels are strongly associated with miscarriage in the first trimester, although nothing is known about putative connections between enzyme levels and miscarriage [9].

This study aimed to determine the relationship between vitamin D and miscarriage by measuring and comparing vitamin D concentration in the serum of women with miscarriage and pregnant women.

## MATERIAL AND METHODS

### Measurements

Twenty-five-OH-VD test kits (Dry Fluorescence Immunoassay) were used for *in-vitro* quantitative measurement of 25-OH-VD (total 25 hydroxyvitamin D) in human serum, plasma, and whole blood. This test was used to assess vitamin D [10].

### Study design and blood samples collection

This study was conducted at Al-Batool Teaching Hospital for Women and Children in Diyala from November/2020 to April/2021. A total of 80 women participated in this research and were divided into two groups. The first group included 40 women with spontaneous abortions, aged between 18–40 years. The second group included 40 pregnant women without previous miscarriages.

About 5 mL of venous blood was collected and dispensed to a plain tube. Blood was left to clot and centrifuged at 3000 rpm for 10 min to obtain sera and moved to another labeled tube to estimate 25 hydroxyvitamin D (25-OH-VD).

### Statistical analysis

The Statistical Package for Social Sciences software version 18 was used to enter and analyze the data. Categorical variables were analyzed using the Chi-square test, and the results were represented using tables and graphs. The significance level for all statistical analyses was set at p=<0.05.

## RESULTS

**Figure 1:**
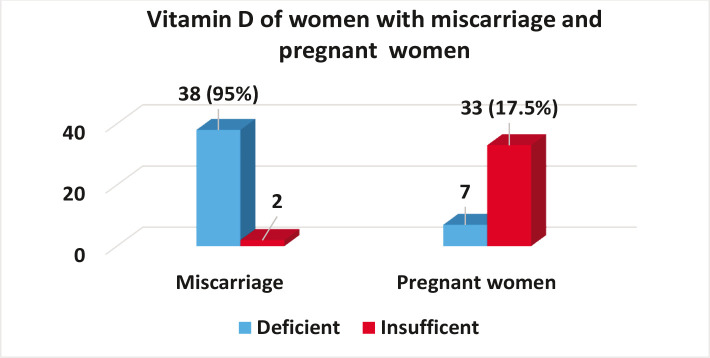
Percentage of vitamin D among women with miscarriage and pregnant women.

The objective of this study was to determine the relationship between vitamin D and spontaneous abortion by assessing the concentration of vitamin D in the serum of women with spontaneous abortion and pregnant women. Out of the 40 samples from women with spontaneous abortion, 38 (95%) had vitamin D deficiency, and 2 (5%) had vitamin D insufficiency. In the group of pregnant women, 7 (17.5%) had vitamin D deficiency *vs*. 33 (82.5%) with vitamin D insufficiency (Figure 1).

### Vitamin D status according to the age of women with spontaneous abortion

**Figure 2:**
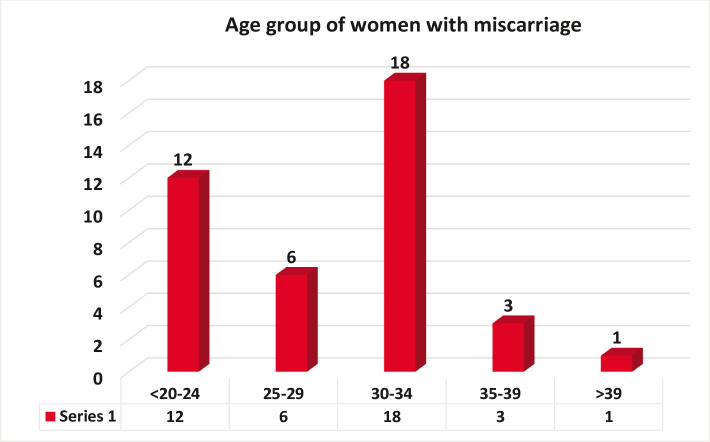
Distribution of age groups and the level of vitamin D among women with spontaneous abortion.

Table 1 and Figure 2 showed that most women with vitamin D deficiency were in the 30–34 years age group (17, 47.5%), and the fewest cases of vitamin D deficiency were observed in the age group of >39 (1, 2.5%).

**Table 1:** Distribution of age groups and levels of vitamin D.

Women with spontaneous abortions			Vitamin D		Total	P-value*	C.S
			Deficient	Insufficient			
Age group	**<20–24**	No.	12	0	12	P>0.05	N.S
		%	30%	0%	30%		
	**25–29**	No.	5	1	6		
		%	12.5%	2.5%	15%		
	**30–34**	No.	17	1	18	P>0.05	N.S
		%	42.5%	2.5%	45%		
	**35–39**	No.	3	0	3		
		%	7.5%	0%	7.5%		
	**>39**	No.	1	0	1		
		%	2.5%	0%	2.5%		
Total		No.	38	2	40		
		%	95%	5%	100%		

* – Independent sample test; N.S – non-significant; C.S – coefficient significance.

### Vitamin D status according to the month of abortion

**Figure 3:**
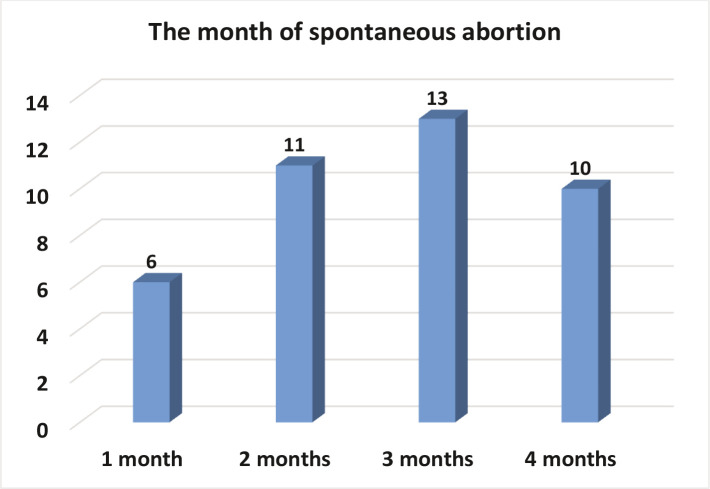
Time of spontaneous abortion (expressed in months) and the level of vitamin D.

Among women with spontaneous abortion at 2 and 3 months of pregnancy, 27.5% and 32.5% had vitamin D deficiency, and 10.25% among women with spontaneous abortion at 4 months (Table 2 and Figure 3).

**Table 2:** Month of spontaneous abortion and levels of vitamin D.

Time of abortion			Vitamin D		Total	P-value*	C.S
			Deficient	Insufficient			
Abortion	**1 month**	No.	6	0	6	P>0.05	N.S
		%	15%	0%	15.0%		
	**2 months**	No.	11	0	11		
		%	27.5%	0%	27.5%		
	**3 months**	No.	11	2	13		
		%	27.5%	5%	32.5%		
	**4 months**	No.	10	0	10		
		%	25%	0%	25%		
Total		No.	38	2	40		
		%	95%	5%	100%		

* – Independent sample test; N.S – non-significant; C.S – coefficient significance.

### Vitamin D status according to age of pregnant women

**Figure 4:**
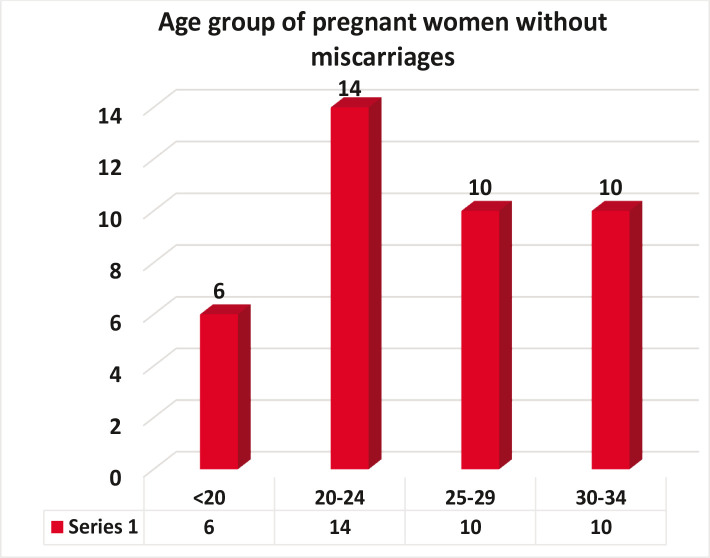
Distribution of age group and levels of vitamin D among pregnant women.

Most cases of pregnant women with insufficient vitamin D levels were under the age group 20–24 years (Table 3 and Figure 4). Furthermore, there were equal cases of insufficient vitamin D levels in the age groups 25–29 and 30–34 (8, 8, 20%, 20%), respectively.

**Table 3:** Distribution of age group and levels of vitamin D among pregnant women.

Pregnant women			Vitamin D		Total	P-value*	C.S
			Deficient	Insufficient			
Age group	**<20**	No.	2	4	6	P>0.05	N.S
		%	5%	10%	15%		
	**20–24**	No.	1	13	14		
		%	2.5%	32.5%	35%		
	**25–29**	No.	2	8	10		
		%	5%	20%	25%		
	**30–34**	No.	2	8	10		
		%	5%	20%	25%		
Total		No.	7	33	40		
		%	17.5%	82.5%	100%		

* – Independent sample test; N.S – non-significant; C.S – coefficient significance.

## DISCUSSION

Differences of vitamin D status according to the age of women with spontaneous abortion was not statistically significant (P>0.05), which agrees with Bodnar (2007) [11] who showed that 33% of women with spontaneous abortion >30 years had lower levels of vitamin D in serum due their lack of milk fortified with vitamin D, and lack of going out and exposure to the sun. Women also used sunscreen, which may prevent the skin from adequate exposure to the sun. There were fewer cases of insufficient Vitamin D observed at 3 months (2, 5%). Statistically, this result was not significant (P>0.05), and this contradicts with Al-Mogbelfound that all Saudi women had low levels of vitamin D throughout the pregnancy [12]. Hypovitaminosis D is quite common in Saudi Arabia, despite the abundance of sunlight. According to our findings, 25(OH)D is associated with miscarriage in the first trimester, contrary to Nagwa El Fadeel Afefy's [13]. That is because there are many distinct mechanisms and factors involved. As a result of different care techniques, the two conditions might be regarded separate disease entities. If losses in the first trimester are linked to 25 (OH) D levels, this might be because 25 (OH) D concentrations are more reflective of serum levels during pregnancy [13].

Differences between vitamin D and age of pregnant women was not significant, and these results were similar to previous studies that reported other factors such as sun exposure, season, skin pigmentation, and nutrition. All these factors affect vitamin D levels in pregnant women. Pregnant women need a lot of important nutritional components during pregnancy because the mother's body consumes all the reserve needed to build the fetus, and therefore the mother needs to compensate for this and supply her body and fetus with what it needs [14].

## CONCLUSIONS

In our study 95% of the women with spontaneous abortions were lacking enough vitamin D, and the largest percentage of those suffering from this deficiency was under the age group 30–34 years. Only 17.5% of pregnant women had vitamin D deficiency. Normal values improve the growth of the fetus and prevents pregnancy complications and abortion. Furthermore, it promotes the growth of blood vessels in the placenta and improves the function of immune cells in the placenta which respond to vitamin D, promoting the healthy development of the fetus.
